# The CXCL13/CXCR5-chemokine axis in neuroinflammation: evidence of CXCR5+CD4 T cell recruitment to CSF

**DOI:** 10.1186/s12987-021-00272-1

**Published:** 2021-08-26

**Authors:** Christine Harrer, Ferdinand Otto, Georg Pilz, Elisabeth Haschke-Becher, Eugen Trinka, Wolfgang Hitzl, Peter Wipfler, Andrea Harrer

**Affiliations:** 1 Department of Neurology, Christian-Doppler-Klinik, Paracelsus Medical University, Ignaz-Harrer-Str 79, 5020 Salzburg, Austria; 2 Department of Laboratory Medicine, Paracelsus Medical University, Landeskrankenhaus, Salzburg, Austria; 3 Research Office, Biostatistics, Paracelsus Medical University, Salzburg, Austria; 4 Department of Ophthalmology and Optometry, Paracelsus Medical University, Salzburg, Austria; 5 Research Program Experimental Ophthalmology and Glaucoma Research, Paracelsus Medical University, Salzburg, Austria

**Keywords:** CSF, CNS, CXCL13, CXCR5, Tfh cells, Neuroinflammation, Flow cytometry

## Abstract

**Background:**

C-X-C chemokine ligand 13 (CXCL13) is frequently elevated in cerebrospinal fluid (CSF) in a variety of inflammatory central nervous system (CNS) diseases, has been detected in meningeal B cell aggregates in brain tissues of multiple sclerosis patients, and proposedly recruits B cells into the inflamed CNS. Besides B cells also follicular helper T (Tfh) cells express the cognate receptor C-X-C chemokine receptor type 5 (CXCR5) and follow CXCL13 gradients in lymphoid tissues. These highly specialized B cell helper T cells are indispensable for B cell responses to infection and vaccination and involved in autoimmune diseases. Phenotypically and functionally related circulating CXCR5+CD4 T cells occur in blood. Their co-recruitment to the inflamed CSF is feasible but unresolved.

**Methods:**

We approached this question with a retrospective study including data of all patients between 2017 and 2019 of whom immune phenotyping data of CXCR5 expression and CSF CXCL13 concentrations were available. Discharge diagnoses and CSF laboratory parameters were retrieved from records. Patients were categorized as pyogenic/aseptic meningoencephalitis (ME, n = 29), neuroimmunological diseases (NIMM, n = 22), and non-inflammatory neurological diseases (NIND, n = 6). ANOVA models and Spearman’s Rank-Order correlation were used for group comparisons and associations of CXCL13 levels with immune phenotyping data.

**Results:**

In fact, intrathecal CXCL13 elevations strongly correlated with CXCR5+CD4 T cell frequencies in the total cohort (p < 0.0001, r = 0.59), and ME (p = 0.003, r = 0.54) and NIMM (p = 0.043, r = 0.44) patients. Moreover, the ratio of CSF-to-peripheral blood (CSF/PB) frequencies of CXCR5+CD4 T cells strongly correlated with CXCL13 levels both in the total cohort (p = 0.001, r = 0.45) and ME subgroup (p = 0.005, r = 0.50), indicating selective accumulation. ME, NIMM and NIND groups differed with regard to CSF cell counts, albumin quotient, intrathecal IgG, CXCL13 elevations and CXCR5+CD4 T cells, which were higher in inflammatory subgroups.

**Conclusion:**

The observed link between intrathecal CXCL13 elevations and CXCR5+CD4 T cell frequencies does not prove but suggests recruitment of possible professional B cell helpers to the inflamed CSF. This highlights CSF CXCR5+CD4 T cells a key target and potential missing link to the poorly understood phenomenon of intrathecal B cell and antibody responses with relevance for infection control, chronic inflammation and CNS autoimmunity.

**Supplementary Information:**

The online version contains supplementary material available at 10.1186/s12987-021-00272-1.

## Introduction

The chemokine C-X-C motif chemokine 13 (CXCL13) has been found elevated in cerebrospinal fluid (CSF) in a variety of inflammatory central nervous system (CNS) diseases [1–4], and proposedly is involved in B cell recruitment to CSF during neuroinflammation [5, 6]. Moreover, the detection of ectopic lymphoid structures (ELS) containing B cells and CXCL13-producing stromal cells in the leptomeninges of secondary progressive multiple sclerosis (MS) patients and CXCL13-immunoreactivity in actively demyelinating lesions [7–9] pointed at a role of CXCL13 also in the structural organization and localization of intrathecal B cell-related immune activities [10].

Both are physiological functions of CXCL13 in secondary lymphoid organs where CXCL13 is crucially involved in the recruitment of B and T lymphocytes and their compartmentalization within B cell follicles of lymph nodes. (SLO) [11, 12]. Initially identified as potent B cell chemoattractant, it soon became evident that also T follicular helper (Tfh) cells, a special subgroup of CD4+ T cells, follow CXCL13 gradients towards the B cell compartments [13]. As B cells, they express the CXCL13-cognate C-X-C chemokine receptor type 5 (CXCR5) which makes them sensitive to CXCL13 signaling, the prerequisite for spatial proximity and productive T cell:B cell interactions. Tfh cells are professional B cell helper T cells and essential for germinal center (GC) formation, B cell differentiation and antibody affinity maturation [13, 14].

Recent research highlighted CXCR5+CD4 T cells in blood as phenotypically and functionally similar circulating counterparts of lymphoid Tfh cells [15, 16]. Circulating CXCR5+CD4 T cells (cTfh) amount to about 10–20% of total CD4 + T cells, comprise Th1-, Th2- and Th17-like subsets differentially regulating B cell responses and and are capable of antigen-specific B cell help with long lasting memory [15–17]. Moreover, so-called recently activated CXCR5+CD4 T cells, identifiable by upregulation of programmed-death receptor-1 (PD1), Ki-67 protein (Ki-67) and ICOS expression, appear in blood in response to specific immune activation post vaccination or during infection [18, 19]. These cells are current hot topic as targets for monitoring and tracking adaptive immune responses to infection and vaccination including HIV, influenza, and lately also Sars COV2 [19–24]. Predominantly Th1-cTfh, their frequencies in blood positively correlate with protective antibody responses [17, 19]. Shifts towards proinflammatory Th17-cTfh cells, in contrast, have been associated with cancer progression and autoimmune disease including MS and myasthenia gravis [15, 25, 26].

CXCR5+CD4 T cells also occur in normal CSF at frequencies similar to peripheral blood [9]. First described in 2006, consequential to the detection of CXCL13 in MS brain tissues, they were neglected for several years. Up-to-date knowledge about the phenotypic and functional diversity of cTfh cells, surface expression of the CXCL13-cognate receptor CXCR5, their mere presence during normal CNS immune surveillance, and the capacity of human (in contrast to murine) Tfh cells to produce CXCL13 themselves strongly support a role in CNS immunity and particular relevance in neuroinflammation with intrathecal CXCL13 elevations and B cell activity.

We thus set out and retrospectively analyzed patient data to learn whether an association between intrathecal CXCL13 elevations and occurrence of CXCR5+CD4 T cells in the inflamed CSF could be established. In light of their potential B cell helper capacities, such evidence would highlight them as relevant targets for further research to improve our understanding of intrathecal B cell and antibody responses to neuroinfection and immune-mediated CNS diseases.

## Methods

### Patients

The study included 57 patients admitted to the Department of Neurology of the Paracelsus Medical University (PMU) in Salzburg, Austria between August 2017 and December 2019 who had undergone lumbar puncture for diagnostic purposes and of whom immune phenotyping data of CXCR5 expression and concentrations of CSF CXCL13 were available. CSF parameters, immune phenotyping results, CXCL13 concentrations and discharge diagnosis were retrospectively collected from chart records.

Based on discharge diagnosis, patients were clinically assigned into three groups: pyogenic & aseptic meningoencephalitis (ME), neuroimmunological disease (NIMM), and non-inflammatory neurological disease (NIND). Diagnoses in the ME group (n = 29) included bacterial meningoencephalitis (n = 2), epidural abscess (n = 1) , tick-borne meningoencephalitis (n = 1), Varicella-Zoster-Virus (VZV) meningoencephalitis (n = 5) Epstein-Barr virus (EBV) meningoencephalitis (n = 3), Influenza B virus meningoencephalitis (n = 1), HIV meningoencephalitis (n = 1), aseptic meningoencephalitis of unknown cause (n = 8), and lyme neuroborreliosis (LNB, n = 7). Diagnoses in the neuroimmunological disease group (n = 22) included multiple sclerosis (MS, n = 8, all but one (natalizumab) untreated), autoimmune encephalitis (AIE) or myelitis (n = 6), neuromyelitis optica (NMO, n = 2), chronic lymphocytic inflammation with pontine perivascular enhancement responsive to steroids (CLIPPERS, n = 1), neurosarkoidosis (n = 1), steroid responsive encephalopathy associated with autoimmune thyroiditis (SREAT, n = 1), and immunological encephalitis not further classifiable (n = 3). Diagnoses in the NIND group (n = 6) included polyneuropathy (n = 1), narcolepsy (n = 1), dementia (n = 1), back pain (n = 1), astrocytoma (n = 1), and epilepsy (n = 1).

The responsible ethics committee of the country of Salzburg consented to the study (415-E/2218/10–2020).

### Standard diagnostic CSF and flow cytometry analysis

**Fig. 1:**
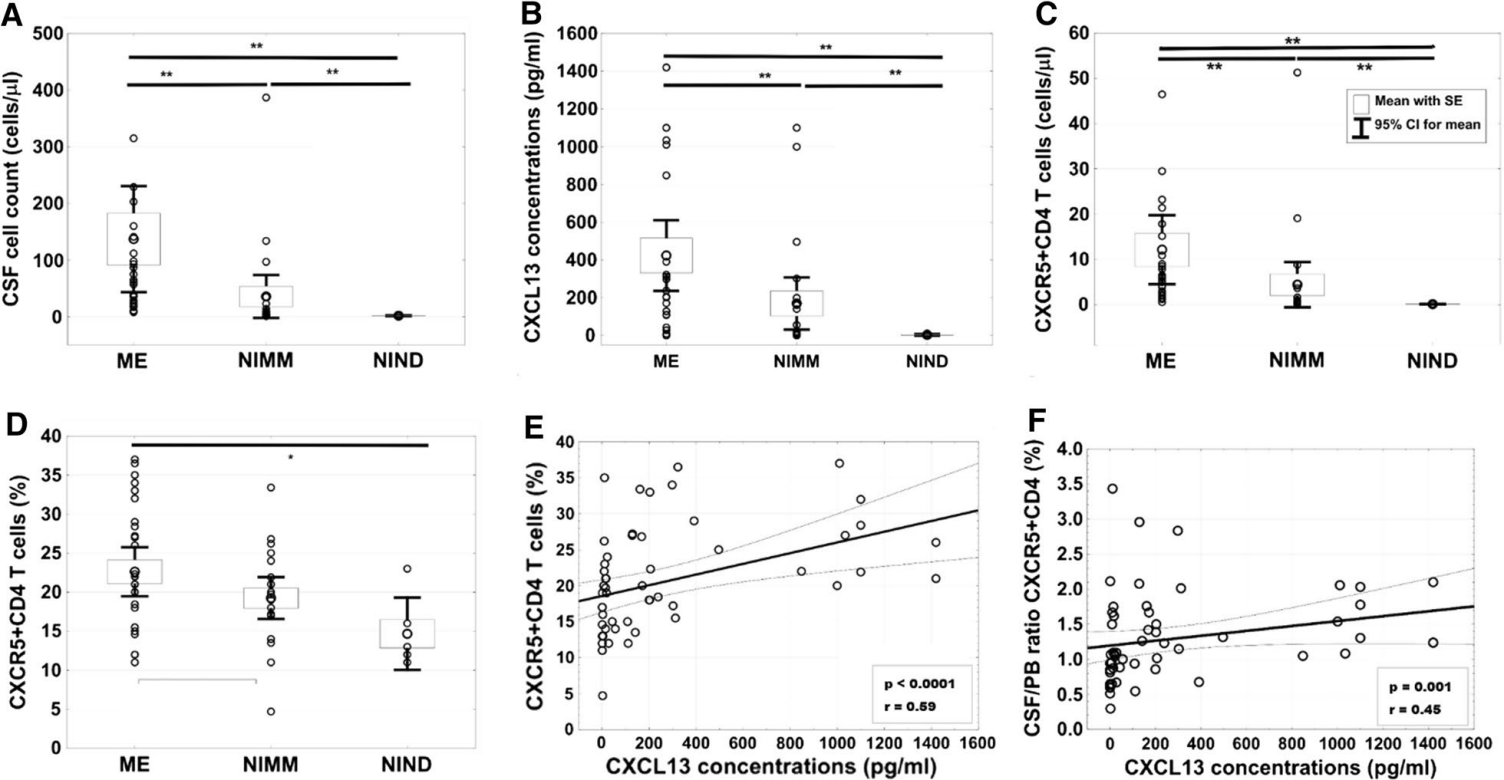
Graphical results of group comparisons and correlation analyses. Plots A-D illustrate comparisons of CSF cell count (**A**), CXCL13 levels (**B**), CXCR5+CD4 T cell numbers (**C**) and CXCR5+CD4 T cell frequencies (**D**) between ME (n = 29), NIMM (n = 22), and NIND (n = 6) subgroups. Association of CSF CXCL13 levels with CXCR5+CD4 T cell frequencies and the CSF/peripheral blood ratio of CXCR5+CD4 T cell frequencies of the total cohort (n = 57 and n = 49 respectively) are shown in plots E and F. ANOVA models were used for group comparisons (**A**, **B**) and Spearman’s Correlation Coefficient test for correlation analysis (**E**, **F**). Boxes visualize means with standard error of the mean (SE). Abbreviations: CSF, cerebrospinal fluid; ME, meningoencephalitis; NIMM, neuroimmunological disease; NIND, non-inflammatory neurological disease; PB, peripheral blood; * = p < 0.05; ** = p < 0.01

CSF laboratory analyses were performed at the Department of Laboratory Medicine of the PMU (certified by the German Society of CSF Diagnostics and Clinical Neurochemistry). CSF cells were enumerated using the Fuchs Rosenthal Counting chamber, IgG and albumin concentrations of CSF and serum samples were measured by immunonephelometry and used to calculate the CSF/serum quotients for albumin (QAlb) as measure for blood-CSF barrier integrity and for IgG (QIgG). Intrathecal IgG synthesis was quantified as the relative intrathecal IgG fraction (% IgG) using the CSF/serum quotient diagrams according to Reiber [27]. CSF CXCL13 concentrations were analysed by enzyme-linked immunorsorbent assay (ELISA) (Euroimmun, Lübeck, Germany). CSF cells were characterized by flow cytometry using in house standard protocols previously reported [28] and summarized in the supplements, which also include a figure illustrating the gating strategy in CSF and EDTA whole blood (Additional file and Figure 1).

### Statistical analysis

Data consistency was checked, and data screened for normal, log-normal and gamma distributions by using Kolmogornov Smirnov tests. Very often continuously distributed data deviated severely from normal distributions and therefore generalized linear models with Tweedie distributions were used to compare groups. Additionally, ANOVA models were used to compare normal distributed variables. The logarithm was used as link function. Chi-Squared Test (categorical variables) was used for group comparisons (ME vs NIMM vs NIND). Spearman’s Rank-Order correlation was used to test the relation of CXCL13 levels with routine CSF parameters and immune phenotyping results. Data of CXCL13 ELISA below the lower limit were arbitrarily set to half the limit and values above the upper limit of quantification were set to twice the limit in analogy to Markowicz et al. [29]. All reported tests were two-sided, and p-values < 0.05 were considered as statistically significant. All statistical analyses and illustrations were done by use of STATISTICA 13 (Hill, T. & Lewicki, P. Statistics: methods and applications. SlatSoft, Tulsa,OK).

## Results

### Patient categorization

This study included 57 patients (46% female) with a median age of 52 years (range 20–88) categorized according to discharge diagnosis as pyogenic & aseptic ME (n = 29; 51%), NIMM n = 22; 39%), and NIND (n = 6; 10%). Groups did not differ with regard to age and gender distribution. Table 1 summarizes baseline characteristics, CSF parameters and immune cell distribution of the total cohort and within subgroups.

**Table 1:** Overview of baseline characteristics, standard CSF parameters and phenotyping results

	Total N = 57	ME n = 29	NIMM n = 22	NIND n = 6	p_1_ Global Test	p_2_ ME vs. NIMM
Age median (range)	52 (20;88)	59 (20;88)	47 (21;75)	49 (32;78)	0.70^a^	^–^
Female n (%)	26 (45.6)	12 (41.4)	12 (54.5)	2 (33.3)	0.53^b^	–
Routine CSF Parameters:						
Cells/µl mean (SD)	83.8 (190.0)	137.1 (246.0)	35.8 (85.2)	1.83 (0.75)	** < 0.0001**^**c**^	** < 0.0001**^**c**^
QAlb mean (SD)	16.8 (21.7)	23.0 (26.5)	11.5 (14.4)	6.15 (1.65)	** < 0.001**^**c**^	**0.005**^**c**^
Intrathecal IgG synthesis (P/N)	17/40	6/23	11/11	0/6	**0.020**^**b**^	0.63^b^
→% IgG mean (SD)	7.30 (15.5)	4.93 (15.0)	12.4 (17.2)	0.00 (0.00)	**0.009**^**c**^	0.14^c^
CXCL13 pg/ml mean (SD)	281.7 (425.4)	423.8 (492.2)	170.3 (310.8)	3.25 (4.08)	** < 0.0001**^**c**^	**0.002**^**c**^

Immune cell phenotyping	%	%	%	%	p_1_	p_2_
CD19 + mean (SD)	4.68 (5.96)	5.28 (6.63)	4.25 (4.67)	3.37 (7.43)	0.54^c^	–
CD3+CD4 mean (SD)	61.7 (12.8)	59.3 (11.3)	66.6 (11.7)	55.7 (18.7)	0.05^c^	–
→CXCR5+CD4 mean (SD)	20.5 (7.46)	22.6 (8.22)	19.3 (6.01)	14.7 (4.41)	**0.020**^**c**^	0.10^c^
CD3 + CD8 mean (SD)	25.6 (10.8)	26.0 (10.5)	22.9 (9.86)	33.7 (12.6)	0.08^c^	0.25^c^
→CXCR5+CD8 mean (SD)	12.7 (9.27)	14.9 (9.96)	10.9 (7.20)	8.90 (11.4)	0.13^c^	–

	Cells/µl	Cells/µl	Cells/µl	Cells/µl	p_1_	p_2_
CD19+ mean (SD)	2.25 (4.05)	3.61 (5.11)	1.06 (1.84)	0.05 (0.12)	** < 0.0001**^**c**^	** < 0.001**^**c**^
CD3 + CD4 mean (SD)	31.8 (56.1)	46.5 (58.9)	20.9 (55.4)	0.64 (0.39)	** < 0.0001**^**c**^	**0.006**^**c**^
→CXCR5+CD4 mean (SD)	7.88 (16.4)	12.1 (20.0)	4.40 (11.3)	0.09 (0.06)	** < 0.0001**^**c**^	** < 0.001**^**c**^
CD3+CD8 mean (SD)	12.4 (20.7)	18.5 (22.6)	7.48 (18.53)	0.40 (0.28)	** < 0.0001**^**c**^	**0.002**^**c**^
→CXCR5+CD8 mean (SD)	1.83 (3.10)	3.00 (3.74)	0.77 (1.74)	0.05 (0.08)	** < 0.0001**^**c**^	** < 0.0001**^**c**^

Bold values represent significant *p* values

CD19+, B cells; CXCR5+CD4, CXCR5+CD4 T cells; CXCR5+CD8, CXCR5+CD8 T cells; CSF, cerebrospinal fluid; CSF %IgG, intrathecal IgG synthesis in percent; ME, meningoencephalitis; NIMM, neuroimmunological disease; NIND, non-inflammatory neurological disease; P/N, positive/negative; QAlb, CSF/Serum Albumin Quotient

^a^ANOVA

^b^Chi Square

^c^Generalized linear model with Tweedie distributions

Expectedly, the two inflammatory subgroups showed higher CSF cell-counts (p < 0.0001, Fig. 1A), QAlb (p < 0.001), intrathecal IgG synthesis (p = 0.02) and CXCL13 levels (p < 0.0001, Fig. 1B) than NIND patients. In line with higher CSF cell counts, they also showed higher absolute numbers of CD4+ and CD8+ T cells, corresponding CXCR5+CD4 (Fig. 1C) and CXCR5+CD8 T cell subsets, and of CD19+ B cells. Analyzing the immune cell composition revealed a global difference toward higher frequencies of CXCR5+CD4 T cells in the inflammatory subgroups compared to NIND (p < 0.02; Fig. 1D).

Comparing the two inflammatory subgroups showed higher CSF cell counts (p < 0.0001), QAlb (p = 0.005), and CXCL13 levels (p = 0.002) in ME compared to NIMM patients, and corresponding to higher cell counts, higher absolute numbers of all five investigated lymphocyte subpopulations and subsets (Table 1).

### CXCL13 elevations versus CXCR5-expression in CSF

The central question of this study was whether occurrence of immune cells expressing cognate CXCR5, in particular CXCR5+CD4 T cells, was associated to CXCL13 concentrations in CSF (Table 2).

**Table 2:** Correlation analysis results

	Total N = 57		ME n = 29		ME w/o LNB n = 22		NIMM n = 22	
	p-value	r-value	p-value	r-value	p-value	r-value	p-value	r-value
CSF CXCL13 levels vs. Routine CSF Parameters:								
Cell Count	** < 0.0001**	0.69	**0.002**	0.55	** < 0.001**	0.69	**0.004**	0.59
QAlb	** < 0.0001**	0.54	**0.039**	0.39	0.05	0.42	**0.048**	0.43
CSF % IgG	0.83	−0.03	0.11	−0.30	0.27	−0.25	0.23	0.27
CSF CXCL13 levels vs Immune Cell Phenotypes (%):								
CD19+	**0.004**	0.38	0.57	0.11	0.64	0.10	**0.005**	0.57
CD3+CD4	0.57	−0.08	0.79	−0.05	0.89	0.03	0.90	0.03
→ CXCR5+CD4	** < 0.0001**	0.59	**0.003**	0.54	**0.001**	0.65	**0.043**	0.44
CD3 +CD8	0.06	−0.25	0.36	−0.18	0.40	−0.19	0.09	−0.37
→ CXCR5+CD8	**0.012**	0.33	0.50	0.13	0.35	0.21	0.32	0.23
CD19+vs. CXCR5+CD4 (%)	** < 0.001**	0.44	0.16	0.27	0.94	−0.02	**0.030**	0.46
CSF CXCL13 vs. immune cell phenotypes (cells/µl):								
CD19+	** < 0.0001**	0.63	0.06	0.36	**0.035**	0.45	** < 0.001**	0.69
CD3+CD4	** < 0.0001**	0.64	**0.025**	0.42	**0.007**	0.56	**0.005**	0.58
→ CXCR5+CD4	** < 0.0001**	0.70	**0.001**	0.58	** < 0.001**	0.68	** < 0.01**	0.60
CD3+CD8	** < 0.0001**	0.61	**0.044**	0.38	**0.021**	0.49	**0.040**	0.44
→ CXCR5+CD8	** < 0.0001**	0.62	**0.048**	0.37	**0.021**	0.49	**0.028**	0.47

	Total N = 49		ME n = 26		ME w/o LNB n = 21		NIMM n = 19	
	p-value	r-value	p-value	r-value	p-value	r-value	p-value	r-value
CSF CXCL13 vs. CSF/PB ratio of immune cell phenotypes (%)								
CSF/PB ratio CD19+	0.10	0.24	0.63	0.09	0.92	−0.02	**0.039**	0.48
CSF/PB ratio CXCR5+CD4	**0.001**	0.45	**0.005**	0.50	**0.010**	0.55	0.18	0.32
CSF/PB ratio CXCR5+CD8	0.17	0.20	0.99	−0.00	0.65	−0.10	0.29	0.25

Bold values represent significant *p* values

All calculations via Spearman Correlation and a p-value < 0.05 considered significant

CSF, cerebrospinal fluid; CSF % IgG, intrathecal IgG synthesis in percent; LNB, Lyme Neuroborreliosis; ME, meningoencephalitis; NIMM, neuroimmunological disease; NIND, non-inflammatory neurological disease; PB, peripheral blood; QAlb, CSF/Serum Albumin Quotient; w/o, without

In fact, correlation analysis of the total cohort revealed positive associations between CXCL13 elevations and percentages of all three CXCR5-expressing lymphocyte populations (%CD19+ B cells: p = 0.004, r = 0.38; %CXCR5+CD4 T cells: p < 0.0001, r = 0.59; %CXCR5+CD8 T cells: p = 0.012, r = 0.33) but not with total CD4 + and CD8+ T cells. The strongest association was between CXCL13 concentrations and CXCR5+CD4 T cell frequencies (Fig. 1E), which remained significant in subgroup analysis (ME: p = 0.003, r = 0.54; NIMM: p = 0.043, r = 0.44) and was even more pronounced upon excluding LNB cases (ME w/o LNB: p = 0.001; r = 0.65). Subgroup analysis further revealed that the association between intrathecal CXCL13 elevations and CD19+ B cell frequencies apparently stemmed from the NIMM subgroup (p = 0.005, r = 0.57). In addition, also CXCR5+CD4 T cell frequencies correlated with CD19+ B cell frequencies in the total cohort (p < 0.001, r = 0.44) and NIMM subgroup (p = 0.030, r = 46).

Correlating CXCL13 concentrations with absolute cell numbers showed positive associations across all five lymphocyte populations/subsets in the total cohort and inflammatory subgroups, which were in accordance with the strong correlation of intrathecal CXCL13 levels and CSF cell counts (total cohort: p < 0.0001; r = 0.69; ME: p = 0.002; r = 0.55; ME w/o LNB: p < 0.001; r = 0.69; and NIMM: p = 0.004; r = 0.59).

CXCL13 levels furthermore correlated with QAlb in the total cohort (p < 0.0001; r = 0.54) and ME (p = 0.039; r = 0.39) and NIMM (p = 0.048; r = 0.43) subgroups, but not with intrathecal IgG synthesis (Table 2).

### The CSF/peripheral blood ratio of CXCR5 expressing immune cells and CSF CXCL13

We next certainly were interested whether intrathecal CXCL13 also correlated with the CSF-to-peripheral blood (PB)-ratio of CXCR5 + T and B cell frequencies to support a possible function in recruitment to the CNS. Corroborating the above findings, we found an association of intrathecal CXCL13 concentrations with the CSF/PB CXCR5+CD4 T cell ratio for the total cohort (p = 0.001; r = 0.45; Fig. 1F) and ME subgroup (p = 0.005; r = 0.50), which again was independent of LNB cases (ME w/o LNB: p = 0.010; r = 0.55), and of intrathecal CXCL13 with the CSF/PB CD19+ B cell ratio for the NIMM subgroup (p = 0.039; r = 0.48) (Table 2).

This analysis was performed with data of 49 patients because two patients had incomplete blood immune phenotyping data, two had chronic lymphocytic leukemia with exorbitant high peripheral blood B cell frequencies, and four patients had received B cell depleting therapy (rituximab) due to Morbus Wegener (last dose in 2013 and peripheral blood B cell counts not recovered several years later), AIE, NMO, and follicular B cell lymphoma. We had to exclude them from analyzing the CSF/blood ratio, because peripheral blood B cell frequencies > 50% would have resulted in misleadingly low ratios and dividing by 0% is not possible.

## Discussion

Based on manifold evidence linking intrathecal CXCL13 elevations with CSF pleocytosis, B cells, plasma cells, and intrathecal immunoglobulin synthesis in neuroinflammation [5, 9, 27, 30], we here set out to clarify a role beyond the proposed B cell recruitment and investigated a link with circulating CXCR5+CD4 T cells. Similar to B cells, these presumptive descendants of professional B cell helper T cells might sense and travel along CXCL13 gradients via its cognate receptor CXCR5.

In fact, our data revealed increased presence of CXCR5+CD4 T cells along with intrathecal CXCL13 elevations in a broad spectrum of inflammatory CNS diseases. Intrathecal CXCR5+CD4 T cell frequencies, absolute numbers of CXCR5+CD4 T cells, and the CSF/PB ratio of CXCR5+CD4 T cell frequencies positively associated to higher CXCL13 levels in CSF, highlighting their preferential recruitment to the inflamed CSF.

Subgroup analysis showed that the positive association of CXCL13 elevations with CXCR5+CD4 T cell frequencies applied in both, ME and NIMM patients and was independent of LNB. The reason why we excluded LNB cases was to ensure that high CXCL13 levels, which are typical for LNB, did not bias results. This sub-analysis accordingly underlined a broader function of CXCL13/CXCR5 signaling in meningoencephalitis and corroborated earlier observations linking persistent CXCL13 elevations with severe disease courses in bacterial and viral CNS infections [31].

Following the argumentation of others regarding the proposed role of intrathecal CXCL13 in the recruitment of B cells [5], our data support a role of CXCL13 elevations in attracting CXCR5+CD4 T cells in both ME and NIMM subgroups and CD19+ B cells in particular in NIMM patients. This is further corroborated by the association of CXCR5+CD4 T cell and CD19+ B cell frequencies. We consider this relevant, because this implies a functional intrathecal CXCL13/CXCR5-chemokine axis facilitating intrathecal proximity of B cells with CXCR5+CD4 T cells, the premise for productive T cell-B cell interactions involving somatic hypermutation, and differentiation to plasma cells [32].

The importance can be seen all the more as we know that B cells not only migrate from blood to CSF but also expand, mature and differentiate within the CNS [33–35]. However, it is still not clear, where exactly and how. An excellent environment might be provided by meningeal ELS with their GC-resembling structures discovered in post-mortem MS brain tissues [10, 36]. Highly organized meningeal ELS, however, rather are the exception than the rule [37]. More immature immune cell aggregates, which often cluster in proximity of meningeal blood vessels are a possible alternative. Especially since a recent animal study using the Theiler’s murine encephalomyelitis virus-induced demyelinating disease (TMEV-IDD) model showed that—similar to observations in peripheral tissue inflammatory cell aggregates sufficed to support intrathecal antibody synthesis [38].

As to the question how B cells may expand, mature and differentiate within the CNS, we propose CXCR5+CD4 T cells as missing link. These so-called “circulating counterparts” of professional B helper Tfh cells, might also be relevant intrathecally as helpers initiating and maintaining antibody-related B cell activities. Our data are no proof but support our thesis of such interactions taking place in the CNS. Latest research in MS using a single cell transcriptomics and gene set enrichment analysis approach came to a similar conclusion. Schafflick and colleagues identified a compartmentalized increase of Tfh cells in CSF, which they proposed to drive the expansion of B lineage cells [39].

Another interesting conclusion touching intrathecal cTfh cells is that of Enose-Akahata and colleagues who characterized the CSF B cell phenotype of human T-lymphotropic virus 1 (HTLV-1) associated myelopathy/tropical spastic paraparesis (HAM/TSP) patients [40]. They reported intrathecal virus-specific antibodies against HTLV-1 antigens Gag and Tax in CSF of HAM/TSP patients but decreased frequencies of Tfh-like cells compared to controls. Of note, HAM/TSP patients are known to develop anti-neuronal antibodies that cross-react with Gag and Tax [41, 42]. As an optimal antibody response requires sufficient Tfh cell numbers for affinity-matured antibodies [43], they concluded that the observed imbalance of intrathecal cTfh cells may have conferred susceptibility to chronic inflammation and autoimmune CNS disease [40].

Limitations of our study are the small patient number, retrospective study-design, and the resultant heterogeneous patient population regarding underlying diseases, causative pathogens, disease stage, disease course, comorbidities, and medication. On the other hand, one might rate the same limitations as strength, since the link between intrathecal CXCL13 levels and CXCR5+CD4 T cells was evident despite small group sizes, patient heterogeneities, and possibly medication-associated decreases of CSF CXCL13 concentrations in response to anti-inflammatory [44, 45], B cell depleting [46] and antibiotic drugs [3]. Moreover, main results are in line with other studies [3, 5, 9], as our data showed a strong association of CXCL13 elevations with CSF pleocytosis, which remained significant when broken down into ME, ME without LNB, and NIMM subgroups, and a positive association with B cell frequencies in NIMM patients. Further prospective studies with larger, more homogeneous patient groups and a deep immune profiling of CSF CXCR5+CD4 T cell phenotypes, subset composition and activation states certainly are warranted and of great interest. They furthermore will allow clues whether observed links between intrathecal CXCL13 levels and CXCR5+CD4 T cells actually relate to their recruitment to CSF or local proliferation or whether CXCR5+CD4 T cells themselves produce and contribute to intrathecal CXCL13 elevations.

## Conclusion

Summing up, we found a clear link between intrathecal CXCL13 elevations and CXCR5+CD4 T cells in particular in meningoencephalitis patients and CXCR5+CD4 T cell correlating with B cell frequencies in neuroimmunological disorders. The sample size is small, but the observation biologically meaningful. In light of their potential B cell helper capacities, the finding suggests intrathecal CXCL13 elevations facilitating compartmentalized proximity and possible productive T cell:B cell interactions in a broad spectrum of inflammatory CNS diseases. We thus propose investigating the intrathecal CXCL13/CXCR5-chemokine axis and CXCR5+CD4 T cell repertoire key to enhance our understanding about B cell activities and intrathecal antibody production for immune defense in CNS infection and immune pathogenic processes leading to chronic inflammation and autoimmune CNS disorders.

## Supplementary Information

**Additional file 1. ** Detailed information about the flow cytometry protocols for processing of CSF and EDTA whole blood and illustration.

## Data Availability

The datasets analyzed during the current study are available from the corresponding author on request.

## Abbreviations

AIE: Autoimmune encephalitis
CNS: Central nervous system
CSF: Cerebrospinal fluid
cTfh: Circulating follicular T helper cells
CXCL13: C-X-C chemokine ligand 13
CXCR5: C-X-C chemokine receptor type 5
ECD: Phycoerythrin Texas Red
ELS: Ectopic lymphoid structures
GC: Germinal center
HAM/TSP: HTLV-1 associated myelopathy/tropical spastic paraparesis
HTLV1: Human T-lymphotropic virus 1
LNB: Lyme neuroborreliosis
ME: Subgroups pyogenic/aseptic meningoencephalitis
MS: Multiple sclerosis
NIMM: Neuroimmunological disease
NIND: Non-inflammatory neurological disease (NIND)
NMO: Neuromyelitis optica
PB: Peripheral blood
QAlb: CSF/serum quotients for albumin
QIgG: CSF/serum quotients for IgG
SLO: Secondary lymphoid organs
SREAT: Steroid responsive encephalopathy associated with autoimmune thyroiditis
Tfh: T follicular helper
VZV: Varicella-Zoster-Virus
