# Supplementary material for: The CXCL13/CXCR5-chemokine axis in neuroinflammation: evidence of CXCR5+CD4 T cell recruitment to CSF
Source: Fluids Barriers CNS. 2021 Aug 26;18:40. doi: 10.1186/s12987-021-00272-1 (PMC8390062; doi:10.1186/s12987-021-00272-1)
Supplement: Supplementary file 1 — Additional file 1. Detailed information about the flow cytometry protocols for processing of CSF and EDTA whole blood and illustration. [file 12987_2021_272_MOESM1_ESM.docx]

**Additional file 1**

**Flow cytometry Protocols for CSF and EDTA whole blood cells**

CSF cells were collected by centrifugation (10 minutes, 270 g, 4°C) within 1 hour after lumbar puncture, resuspended in precooled (4-6%C) flow cytometry (FC)-buffer (phosphate buffered saline (PBS) supplemented with 2% fetal calf serum and 4 mM EDTA, sterile-filtered) and stained (25 minutes, dark, 4-6°C) with saturating amounts premixed detection antibodies at 50 µl volumes. Cells were washed once with 1 ml FC-buffer (centrifugation at 270 g, 7 minutes, 4°C), resuspended in 250 µl FC-buffer, and immediately subjected to flow cytometry.

Paired whole blood was processed in parallel by adding saturating amounts of the identical premixed antibodies into a staining volume of 100 µl (15 minutes, dark, room temperature). Erythrocytes were lysed for 10 minutes by adding 1 ml of VersaLyse (Beckman Coulter, Vienna, Austria), washed twice with 2-3 ml FC-buffer (270 g, 7 minutes, room temperature), fixed in 0.1% para-formaldehyde in PBS and kept in the refrigerator (4-6°C, dark) until flow cytometry subsequent to CSF samples. The monoclonal detection antibodies used were anti-CD3-phycoerythrin Texas Red conjugate (CD3-ECD; clone UCTH1), anti-CD4-fluorescein isothiocyanate (CD4-FITC, clone 13B.2), anti-CD8-phycoerytrhin cyanin 5.1 conjugate (CD8-PC5; clone B9.11), anti-CD19-phycoerythrin cyanin 7 conjugate (CD19-PC7; clone J3-119), anti-CD20-phycoerythrin (CD20-PE; clone B9E9), anti-CD45-FITC (clone J33), anti-CD45-ECD (clone J33), anti-CD185(CXCR5)- PE (clone MU5UBEE). Except for CXCR5-PE (ThermoFisher Scientific, Vienna, Austria) all other antibodies were from Beckman Coulter.

Cells were acquired on a Cytomics FC500, analyzed using Kaluza software (both from Beckman Coulter) and gated according to forward scatter (FS)/side scatter (SS) properties and CD45-expression for determining the parent lymphocyte gate for analyzing T cell subpopulations and B cells. T and B cells were analyzed from separate tubes. CD19+ B cells were gated either as CD19+CD20+ population out of a CD45-FITC gate and/or as CD19+ population out of a CD45-ECD gate, dependent on the main underlying differential diagnostic question and number of tubes with regard to CSF cell count.

**Figure S1.** Gating Strategy


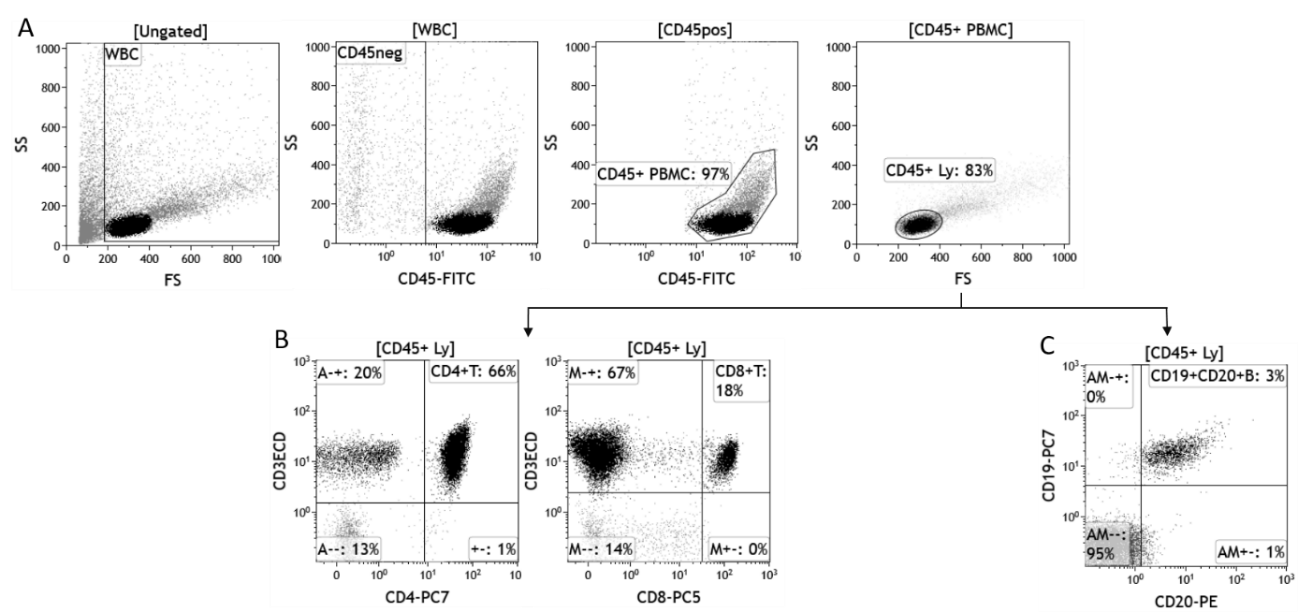


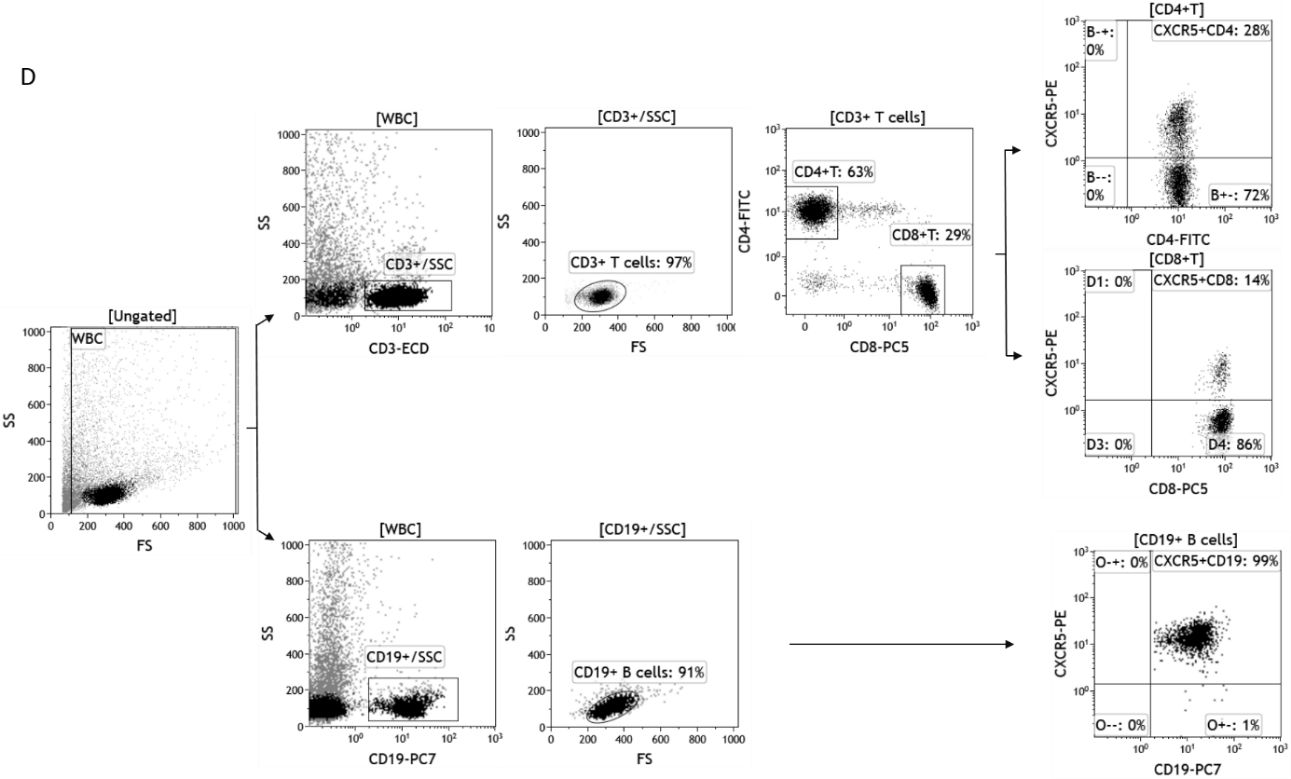


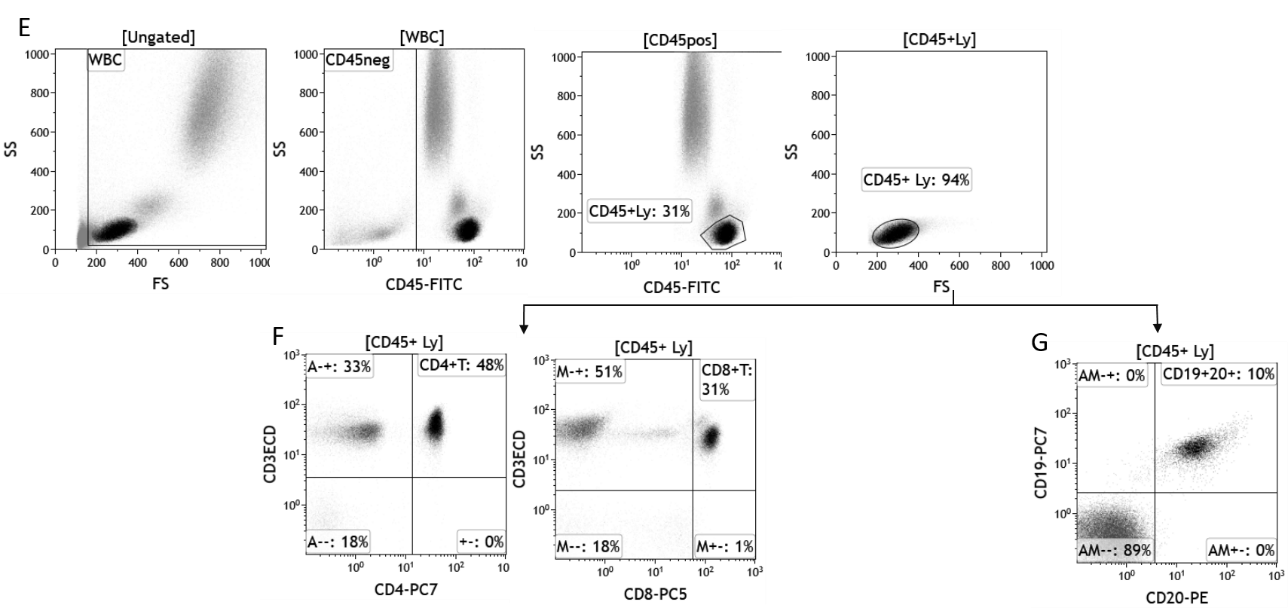
**
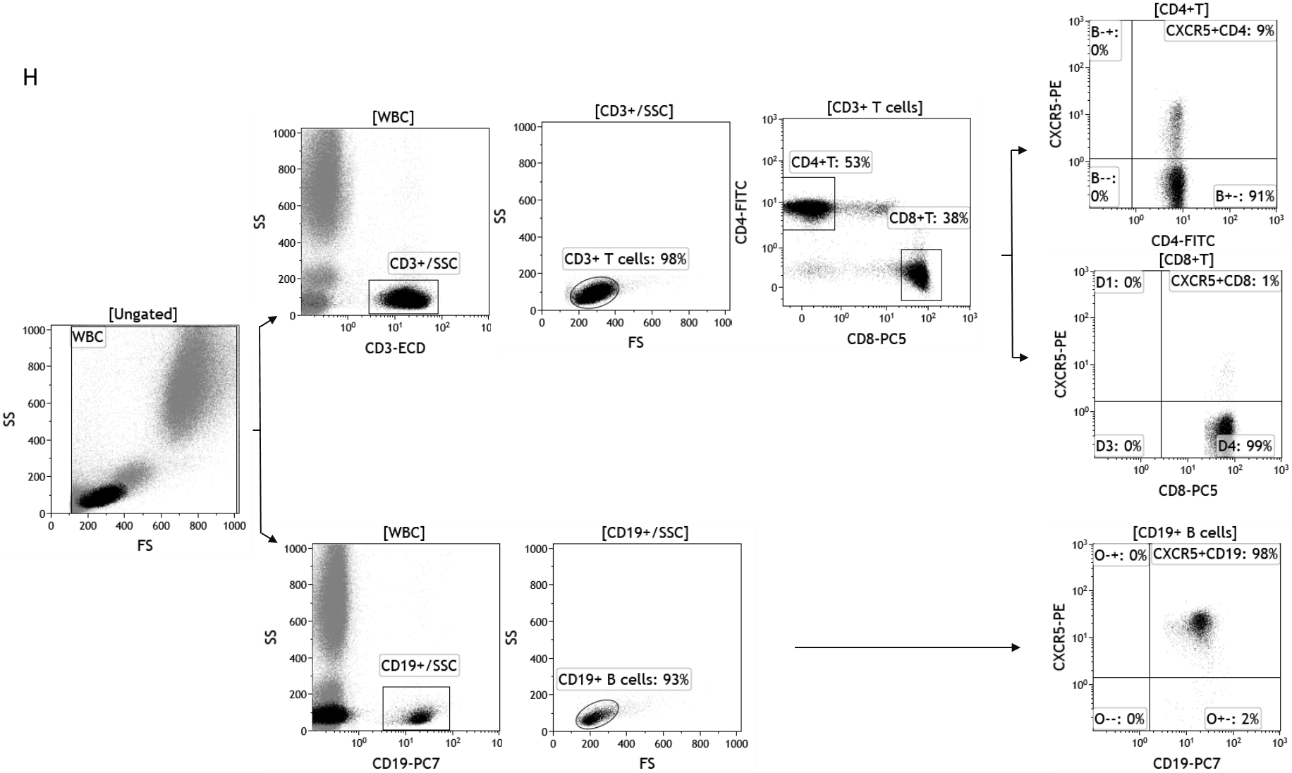
**

**Figure S1.** Gating strategy of CSF and peripheral blood cells. In (A) the initial WBC gate according to FS/SS properties for removal of debris, positive selection of CD45+ PBMC, and back gating of CD45+ lymphocytes is shown. The CD45+ Ly gate served as basis for determination of CD4+ T and CD8+ T cells (B) and CD19+ B cells (C). Analysis of CXCR5 expression is shown in (D) and includes a general WBC gate to remove debris and positive selection of CD3+ T cells and CD4+ and CD8+ T cell subpopulation (upper panel) and B cells (lower panel). Abbreviations: CSF, cerebrospinal fluid; FS, forward scatter; Ly, lymphocytes; SS (SSC), side scatter; PBMC, peripheral blood mononuclear cells; WBC, white blood cells.

Whole blood cells were gated analogous to CSF cells. In (E) the initial WBC gate according to FS/SS properties for removal of debris, positive selection of CD45+ PBMC, and back gating of CD45+ lymphocytes is shown. The CD45+ Ly gate served as basis for determination of CD4+ T and CD8+ T cells (F) and CD19+ B cells (G). Analysis of CXCR5 expression is shown in (H) and includes a general WBC gate to remove debris and positive selection of CD3+ T cells and CD4+ and CD8+ T cell subpopulation (upper panel) and B cells (lower panel). Abbreviations: CSF, cerebrospinal fluid; FS, forward scatter; Ly, lymphocytes; SS (SSC), side scatter; PBMC, peripheral blood mononuclear cells; WBC, white blood cells.
